# Detection and Decontamination of Chronic Wasting Disease Prions during Venison Processing

**DOI:** 10.3201/eid3104.241176

**Published:** 2025-04

**Authors:** Marissa Milstein, Sarah C. Gresch, Marc D. Schwabenlander, Manci Li, Jason C. Bartz, Damani N. Bryant, Peter R. Christenson, Laramie L. Lindsey, Nicole Lurndahl, Sang-Hyun Oh, Gage R. Rowden, Rachel L. Shoemaker, Tiffany M. Wolf, Peter A. Larsen, Stuart S. Lichtenberg

**Affiliations:** University of Minnesota, St. Paul, Minnesota, USA (M. Milstein, S.C. Gresch, M.D. Schwabenlander, M. Li, D.N. Bryant, L.L. Lindsey, N. Lurndahl, G.R. Rowden, R.L. Shoemaker, T.M. Wolf, P.A. Larsen, S.S. Lichtenberg); University of Minnesota, Minneapolis, Minnesota, USA (M. Li, P.R. Christenson, S.-H. Oh); Creighton University, Omaha, Nebraska, USA (J.C. Bartz)

**Keywords:** chronic wasting disease, prions, food safety, RT-QuIC, seeded amplification assay, transmissible spongiform encephalopathy, white-tailed deer, United States

## Abstract

Prion diseases, including chronic wasting disease (CWD), are caused by prions, which are misfolded aggregates of normal cellular prion protein. Prions possess many characteristics that distinguish them from conventional pathogens, in particular, an extraordinary recalcitrance to inactivation and a propensity to avidly bind to surfaces. In middle to late stages of CWD, prions begin accumulating in cervid muscle tissues. Those features collectively create scenarios in which occupational hazards arise for workers processing venison and pose risks to consumers through direct prion exposure through ingestion and cross-contamination of food products. In this study, we demonstrate that steel and plastic surfaces used in venison processing can be directly contaminated with CWD prions and that cross-contamination of CWD-negative venison can occur from equipment that had previously been used with CWD-positive venison. We also show that several decontaminant solutions (commercial bleach and potassium peroxymonosulfate) are efficacious for prion inactivation on those same surfaces.

Chronic wasting disease (CWD) is a fatal prion disease affecting cervids caused by a self-templating, misfolded, and infectious form of the prion protein (PrP^Sc^) (*1*). Since its discovery in the United States in the 1960s (*2*,*3*), CWD has been detected in free-ranging and captive cervid populations in 34 US states and 5 provinces in Canada, as well as Nordic countries and South Korea (*4*). CWD continues to spread in white-tailed deer (*Odocoileus virginanus*), mule deer (*Odocoileus hemionus*), moose (*Alces alces*), and elk (*Cervus canadensis*) populations across the United States and Canada. In the United States alone, >6 million white-tailed deer are harvested annually, many of which are consumed and represent a major source of protein for communities across the country (*5*). A 2017 estimate suggests that as many as 15,000 CWD-positive white-tailed deer are consumed in the United States annually (*6*). This number, however, is likely underestimated given the limitations of existing CWD surveillance programs and venison food donation efforts within CWD-endemic regions.

CWD prions accumulate in tissues during disease pathogenesis and can be detected in the muscle tissue of white-tailed deer (*7*); thus, consuming meat from CWD-positive cervids might expose humans to CWD prions. Scientific and public health communities are concerned about the potential transmission of CWD to humans, particularly through ingestion. The Centers for Disease Control and Prevention acknowledges this risk and recommends reducing risk by testing cervids before consuming meat, processing each animal individually to avoid cross-contamination, and not consuming CWD-positive meat (*8*). No cases of CWD in humans have been confirmed (*9*); however, as new CWD strains are identified (*10*) and more organisms are exposed to PrP^Sc^ (*11*), concerns are growing that the species barrier might be crossed.

When PrP^Sc^ prions are introduced into the environment through natural shedding (*12*,*13*) or carcass decomposition (*14*), they can adsorb to surfaces where they can be detected long after deposition (*15*–*17*). Surface swabbing is an effective CWD detection method for both laboratory settings (*18*) and natural, environmentally exposed surfaces (*19*). Yuan et al. (*18*) highlighted the importance of surface structure for prion recovery, noting that porous surfaces, such as wood, were ineffective for swab-based detection, as opposed to nonporous surfaces, such as glass and stainless steel. Those attributes (e.g., environmental stability, swab detection, surface adsorption) also factor into surface decontamination, because chemical decontaminants must physically contact PrP^Sc^ aggregates for disintegration or other forms of inactivation (*20*–*22*).

Venison processing for human consumption, both in-home and commercial, is an area of potential PrP^Sc^ cross-contamination and direct human exposure. Surfaces, tools, and equipment used for venison processing can be contaminated with PrP^Sc^ from CWD-positive venison (*23*,*24*). Cleaning strategies used during venison processing can vary widely and might not be effective in removing or destroying PrP^Sc^, particularly in unregulated facilities or scenarios (e.g., home butchery, seasonal pop-up processors). Thus, understanding the potential of PrP^Sc^ to more widely enter the food supply through surface contamination and the efficacy of chemical decontamination are clearly needed.

In this study, we examined prion contamination of commonly used meat-processing equipment, including knives, cutting boards, and household-style meat grinders, and the efficacy of decontaminants commonly used in home or commercial processing. In addition, we investigated the cross-contamination of CWD-negative meat after contact with CWD-contaminated processing equipment.

## Methods

**Figure 1:**
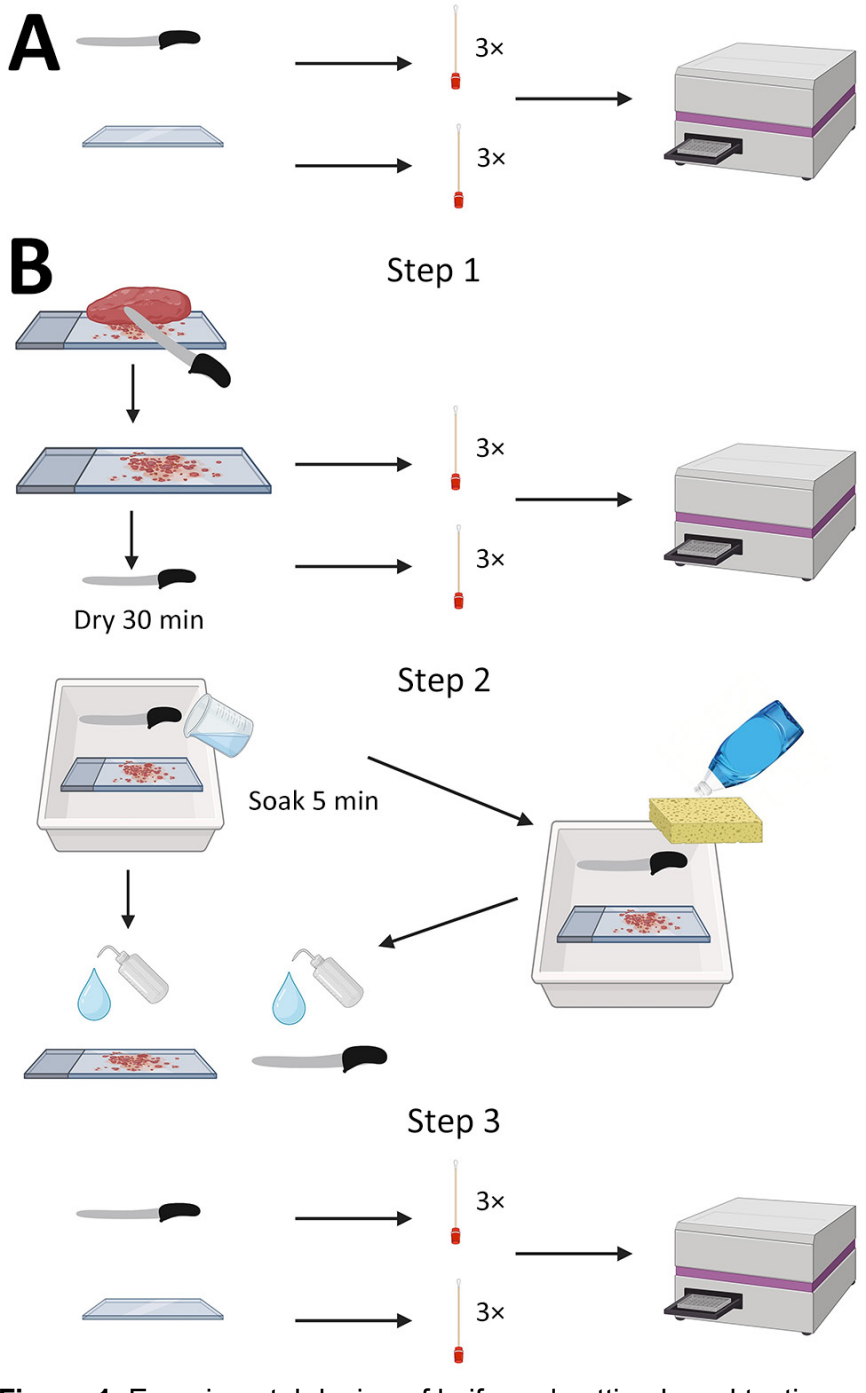
Experimental design of knife and cutting board testing in study of detection and decontamination of chronic wasting disease prions during venison processing. A) For negative control, surfaces were swabbed before use. B) Step 1: chronic wasting disease–negative or chronic wasting disease–positive muscle was cut and surfaces were swabbed. Step 2: surfaces were cleaned. Step 3: surfaces were swabbed again and swabs were tested by real-time quaking-induced conversion. Figure created using BioRender (https://www.biorender.com).

This work consisted of 5 distinct phases. The 5 phases were pilot study, study controls, knife and cutting board, meat cross-contamination, and meat grinder (Table 1, Figure 1; Appendix).

**Table 1:** Overview of study phases and goals in study of detection and decontamination of CWD prions during venison processing*

Study phase	Goal
Pilot study	Determine whether we could recover, detect, and decontaminate prions on common meat-processing surfaces.
Study controls	Confirm that the selected decontaminants did not interfere with the real-time quaking-induced conversion assay and that CWD-positive material could be detected on both stainless steel and cast iron surfaces. Determine whether the decontaminants inhibit seeding activity.
Knife and cutting board	Determine whether prion seeding activity could be detected before and after decontaminating stainless steel knives and cutting boards. Test the efficacy of 5 decontaminants on those surfaces.
Meat cross-contamination	Determine whether CWD-negative meat becomes CWD-positive after passing through a meat grinder that just processed CWD-positive meat.
Meat grinder	Determine whether prion seeding activity could be detected before and after decontaminating stainless steel and cast iron meat grinders. Test the efficacy of 4 decontaminants on the grinders.

*CWD, chronic wasting disease.

### Pilot Study

We conducted a pilot study to determine whether we could recover, detect, and decontaminate prions on common meat-processing surfaces with known CWD-positive samples with different prion loads from white-tailed deer: cerebellum (high prion load) and muscle (low prion load). We conducted control experiments to determine whether the decontaminants could induce or suppress seeding activity during real-time quaking-induced conversion (RT-QuIC) (Appendix). 

### Study Controls

We performed experiments to test whether 5 chosen decontaminants would interfere with the RT-QuIC assay and whether those decontaminants would inhibit prion seeding activity on both stainless steel and cast iron surfaces. Chosen decontaminants were dish soap (Dawn brand; Procter and Gamble, https://dawn-dish.com), Briotech (0.02% hypochlorous acid solution; https://briotechusa.shop), Virkon-S (2% potassium peroxymonosulfate solution; Lanxess AG, https://lanxess.com), and 10% vol/vol (7,500 ppm) and 40% vol/vol (30,000 ppm) commercial bleach solution (7.5% sodium hypochlorite; The Clorox Company, https://www.clorox.com). In addition, we conducted experiments to assess the recovery and detection of CWD prions from stainless steel and cast iron surfaces.

### Knife and Cutting Board

We sought to determine whether prion seeding activity could be detected before and after decontamination on stainless steel knives and polyethylene cutting boards, 2 standard pieces of equipment used in home and commercial meat processing. We designated a knife and cutting board for each of the 5 chosen decontaminants. We collected negative controls by swabbing the knife and cutting board before contact with any muscle samples. 

To test the efficacy of dish soap for prion decontamination, we made 2 cuts through CWD-negative muscle on the cutting board. We immediately swabbed the knife and cutting board after the first cut and left them to dry at room temperature for 30 minutes after the second cut. We filled a tray with dish soap and water according to manufacturer recommendations, scrubbed the knife and cutting board with a sponge (3M, https://www.scotch-brite.com), and then rinsed with low-pressure municipal cold water. We then swabbed the surfaces again. We repeated the experiment using CWD-positive muscle and new materials. We stored all swabs at −80°C until use.

To test the efficacy of Briotech, 2% Virkon-S, 10% vol/vol (7,500 ppm) bleach solution and 40% vol/vol (30,000 ppm) bleach solution, we followed the same procedure as described but did not perform the sponge step. Instead, after drying the polyethylene cutting board and knife for 30 minutes, we soaked the items in the decontaminant solution for 5 minutes. Then, we rinsed the knife and cutting board with water and swabbed them again (Figure 1).

### Meat Cross-Contamination

We passed CWD-positive muscle samples through a meat grinder several times to produce a homogenized pool of CWD-positive muscle, which we then subsampled. We used that pool of CWD-positive material for the meat grinder experiments. We disassembled the grinder, removed the gross material, and swabbed the parts. We then reassembled the grinder, passed CWD-negative muscle through the grinder, and subsampled.

We performed an endpoint titration on the homogenized CWD-positive muscle pool to determine prion load relative to our positive lymph node control. We tested the muscle pool at dilutions from 10^–1^ to 10^–9^. We observed statistically significant seeding activity at 10^–1^ and 10^–2^ dilution and nonsignificant seeding activity at 10^–3^ dilution. We observed no seeding activity in the pool at dilutions 10^–4^ through 10^–9^. Comparatively, our positive lymph node control has historically shown seeding activity through a dilution of 10^–6^.

### Meat Grinder

On the basis of preliminary results demonstrating the efficacy of the decontaminants used in the knife and polyethylene cutting board study, we chose the following 4 decontaminants to be used in experiments using meat grinders: dish soap, Virkon-S, 10% (7,500 ppm) bleach solution, and 40% (30,000 ppm) bleach solution. We used stainless steel (CHOLISM) and cast iron (CucinaPro, https://cucinapro.com) meat grinders with each decontaminant. We disassembled each grinder and swabbed the worm spindle, plate cutter holes, and screw ring threads before contact with CWD-positive muscle. The experiments were designed to mimic home or small-scale commercial meat processing.

#### Cleaning of Grinder after CWD-Positive Homogenate

**Figure 2:**
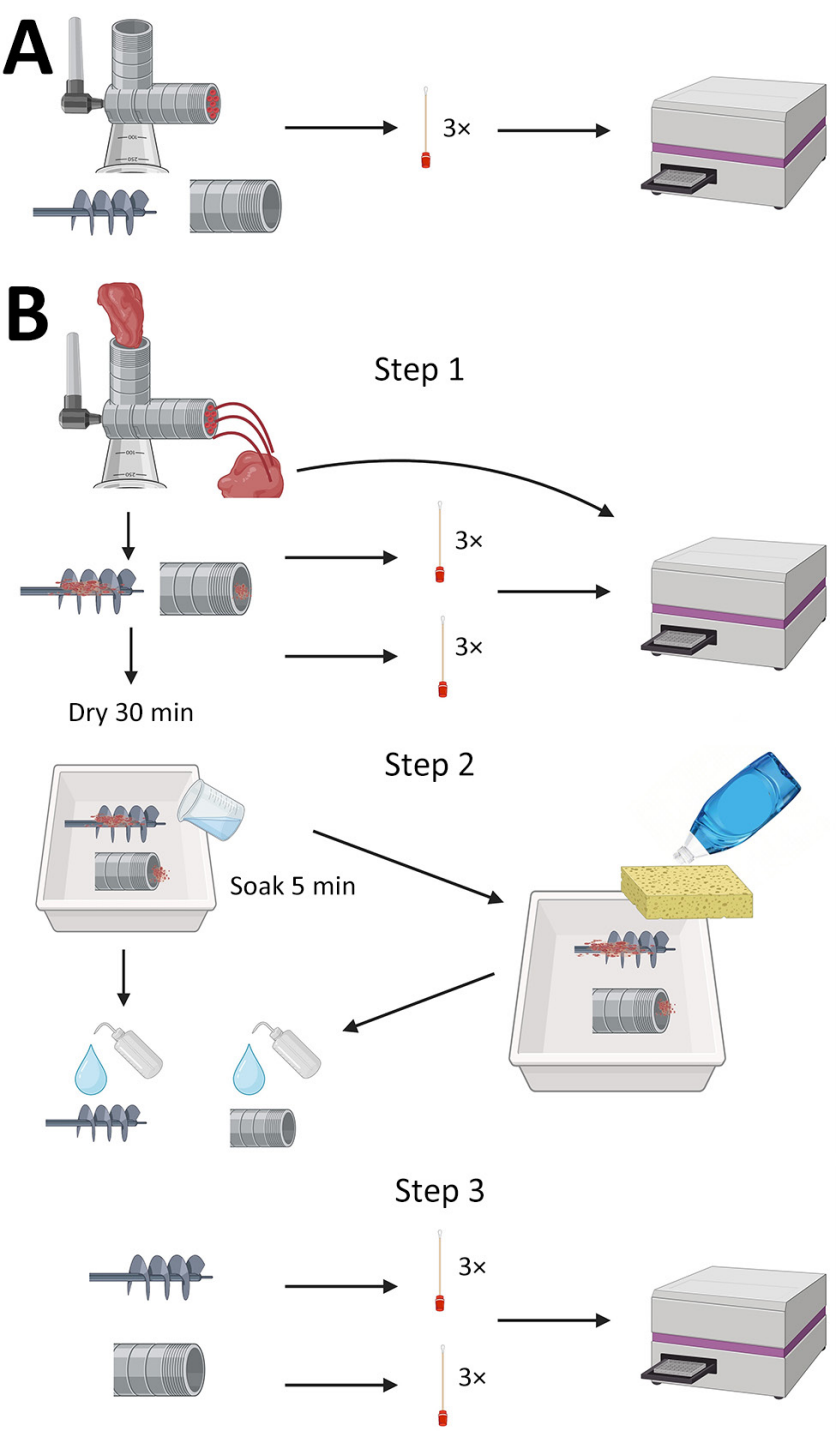
Experimental setup of the meat grinder testing in study of detection and decontamination of chronic wasting disease prions during venison processing. A) For negative control, surfaces were swabbed before use. B) Step 1: chronic wasting disease–negative or–positive muscle was passed through the grinder and surfaces swabbed. Step 2: grinders were disassembled and surfaces were cleaned/decontaminated. Step 3: grinder surfaces were swabbed again, and swabs were tested by real-time quaking-induced conversion. Figure created using BioRender (https://www.biorender.com).

After each grinder was assembled, we passed through CWD-positive homogenate and then allowed the grinder to air dry for 30 minutes. We then disassembled the grinders, removed gross material, and swabbed the grinder parts again. We then placed the parts in a tray with diluted dish soap, used a sponge to wash them, and rinsed them with water. We sampled the sponge and swabbed the grinder parts. For the remaining 3 decontaminants (Virkon-S, 10% [7,500 ppm] bleach, and 40% [30,000 ppm] bleach), we left the grinder parts to soak for 5 minutes (instead of washing with a sponge), rinsed them with low-pressure cold water, and swabbed in the same locations (Figure 2). Swab extraction and processing followed the procedure of Yuan et al. (*19*) with some modifications (Appendix).

### Analysis

Fluorescence readout from the RT-QuIC assay captures the kinetics of amyloid formation in vitro (*25*). Three metrics can be used to describe data generated by RT-QuIC: rate of amyloid formation (RAF) for nucleation, maximum slope (MS) for elongation (*26*,*27*), and maxpoint ratio (MPR) for equilibrium (*28*). We calculated RAF per well as the reciprocal of the time required for fluorescence to reach a threshold of twice the background fluorescence. If a well did not reach the threshold, we assigned a value of zero. We calculated background fluorescence as the average fluorescent reading per well at cycles 2 and 3 to control for any variability in relative fluorescence units (RFU) among wells and to compensate for the greater ThT fluorescence typically found in the first cycle because of viscosity effects. We calculated the slope as the difference between the RFU at the current time position plus 6 cycles (3.75 hours) divided by the time. MPR was calculated as the maximum RFU divided by the background RFU. Using all 3 metrics, genuine amyloid formation of PrP^Sc^ induced by CWD prions in RT-QuIC reactions should generate responses that are significantly different from reactions with no seeding activities.

A member of the research team (M.L.), blinded to sample identity and treatment, analyzed the data generated from the pilot study. Using a 1-tailed Wilcoxon Rank Sum test, with an ɑ-level of 0.05, we compared RAF, MPR, and MS against the negative plate controls. M.L. and unblinded team members (M.M., S.G.) reviewed the results from the pilot data and set the following swab inclusion criteria for the remainder of the study: all 3 metrics (RAF, MPR, MS) must be statistically significantly higher than the negative plate controls to be considered positive, and regardless of dilution level, if a sample meets the first criterion, the sample will be considered positive. We performed all statistical analysis for swab samples in R version 4.3.2 (The R Project for Statistical Computing, https://www.r-project.org).

For all muscle sample analyses, we used an uncorrected Fisher LSD test with a single pooled variance (α level of 0.05). We compared RAF, MPR, and MS against the negative plate controls. All 3 metrics (RAF, MPR, MS) must be statistically significantly higher than the negative plate controls to be considered positive. If a sample met those criteria, it was considered positive regardless of the dilution level. We performed all statistical analyses of muscle samples using GraphPad Prism version 10.0.2 (https://www.graphpad.com).

## Results

### Pilot Study

Multiple swabs demonstrated significant seeding activity in the RT-QuIC assay: knife blade swabs and polyethylene cutting board swabs from cutting CWD-positive muscle and knife blade swabs and cutting board swabs from cutting CWD-positive brain. After soaking the knife and cutting board with 3 dilutions of household bleach (10%, 40%, 100%), all posttreatment swabs (n = 30; 2 or 3 knife swabs and 3 cutting board swabs per dilution, per sample type) were negative by RT-QuIC.

### Study Controls

All negative controls (both knife and polyethylene cutting board) were negative by RT-QuIC for each of the 5 decontaminants after treatment (Tables 2,3). For each of the 5 decontaminants (dish soap, Briotech, Virkon-S, 10% [7,500 ppm] bleach, 40% [30,000 ppm] bleach), all samples had no significant seeding activity when CWD-negative muscle tissue was used during knife and cutting board experiments (Tables 2,3).

**Table 2:** Results of testing of decontaminants in study of detection and decontamination of CWD prions during venison processing

Substance†	Unspiked decontaminant control samples					Decontaminant control samples spiked with CWD-positive homogenate					Decontaminants rinsed then spiked with CWD-positive homogenate			
	No. positive	p value				No. positive	p value				No. positive	p value		
		RAF	MPR	MS			RAF	MPR	MS			RAF	MPR	MS
Dish soap	0/3	1.0000	0.0048	0.5000		**3/3**	**0.0029**	**0.0048**	**0.0048**		**3/3**	**0.0029**	**0.0048**	**0.0048**
		0.0047	0.0048	0.0857			**0.0029**	**0.0048**	**0.0048**			**0.0029**	**0.0048**	**0.0070**
		1.0000	0.6191	0.7619			**0.0029**	**0.0048**	**0.0048**			**0.0029**	**0.0048**	**0.0048**
Briotech	0/3	0.5000	0.0857	0.0857		**3/3**	**0.0029**	**0.0070**	**0.0070**		**3/3**	**0.0029**	**0.0048**	**0.0048**
		0.3593	0.1762	0.1762			**0.0029**	**0.0070**	**0.0070**			**0.0029**	**0.0048**	**0.0048**
		0.9146	0.5429	0.5426			**0.0025**	**0.0048**	**0.0048**			**0.0029**	**0.0048**	**0.0048**
Virkon-S	0/3	0.5000	0.4571	0.3048			**0.0126**	**0.0048**	**0.0048**		**3/3**	**0.0029**	**0.0048**	**0.0048**
		0.5000	0.0333	0.0571		1/3	0.0471	0.0571	0.1962			**0.0029**	**0.0048**	**0.0048**
		0.8463	0.1286	0.1286			0.1537	0.1286	0.7619			**0.0029**	**0.0048**	**0.0048**
10% bleach	0/3	1.0000	0.8714	0.9429		0/3	0.1537	0.3810	0.5847		**3/3**	**0.0029**	**0.0048**	**0.0048**
		1.0000	0.5429	0.9655			1.0000	0.3810	0.9879			**0.0028**	**0.0070**	**0.0070**
		1.0000	0.0333	0.6191			1.0000	0.6191	0.9328			**0.0029**	**0.0070**	**0.0070**
40% bleach	0/3	0.5522	0.5429	0.8038		0/3	1.0000	0.0070	0.9963		**3/3**	**0.0029**	**0.0070**	**0.0070**
		0.5522	0.6952	0.8714			1.0000	0.0070	0.9963			**0.0029**	**0.0070**	**0.0070**
		0.5522	0.3048	0.7619			1.0000	0.0346	0.9789			**0.0029**	**0.0070**	**0.0070**

*Selected decontaminants were tested to determine if they directly interfered with the real-time quaking-induced conversion assay. CWD-positive homogenate was added to surfaces actively being treated, and after rinsing away the decontaminants to assess if they inhibited prion seeding activity. Bold indicates sample replicates found to have statistically significant seeding activity (p<0.05). CWD, chronic wasting disease; MPR, maxpoint ratio; MS, maximum slope; RAF, rate of amyloid formation. †Dish soap, Dawn brand (Procter & Gamble, https://dawn-dish.com); Briotech, 0.02% hypochlorous acid (https://briotechusa.shop); Virkon-S, 2% potassium peroxymonosulfate (Lanxess AG, https://lanxess.com); bleach, 10% vol/vol (7,500 ppm) and 40% vol/vol (30,000 ppm) commercial bleach solutions (7.5% sodium hypochlorite; The Clorox Company, https://www.clorox.com).

**Table 3:** High-level overview of findings of study of detection and decontamination of CWD prions during venison processing*

Study phase	Result†
Pilot study	Prions can be recovered and detected on common meat processing items. 10%, 40%, and 100% bleach were effective at decontaminating meat processing surfaces.
Study controls	The selected decontaminants do not interfere with the assay. Prions could be recovered and detected on surfaces that had processed CWD-positive meat. Surfaces swabbed after processing CWD-negative meat tested negative for seeding activity.
Knife and cutting board	Dish soap removed seeding activity from knife blades but not cutting boards. Briotech removed seeding activity from knives but only partially from cutting boards. Virkon-S, 10% bleach, and 40% bleach removed seeding activity from knives and cutting boards.
Meat cross-contamination	CWD-negative meat was contaminated (5 of 8 samples tested positive) after passing through a grinder that had processed CWD-positive meat ≈5 min earlier.
Meat grinder	Dish soap removed seeding activity from cast iron grinders but not stainless steel. Virkon-S reduced but did not eliminate seeding activity from cast iron and stainless steel grinders. 10% and 40% bleach removed seeding activity from cast iron and stainless steel grinders.

*CWD, chronic wasting disease. †Dish soap, Dawn brand (Procter & Gamble, https://dawn-dish.com); Briotech, 0.02% hypochlorous acid (https://briotechusa.shop); Virkon-S, 2% potassium peroxymonosulfate (Lanxess AG, https://lanxess.com); bleach, 10% vol/vol (7,500 ppm) and 40% vol/vol (30,000 ppm) commercial bleach solutions (7.5% sodium hypochlorite; The Clorox Company, https://www.clorox.com).

### Knife and Cutting Board

#### Dish Soap

**Figure 3:**
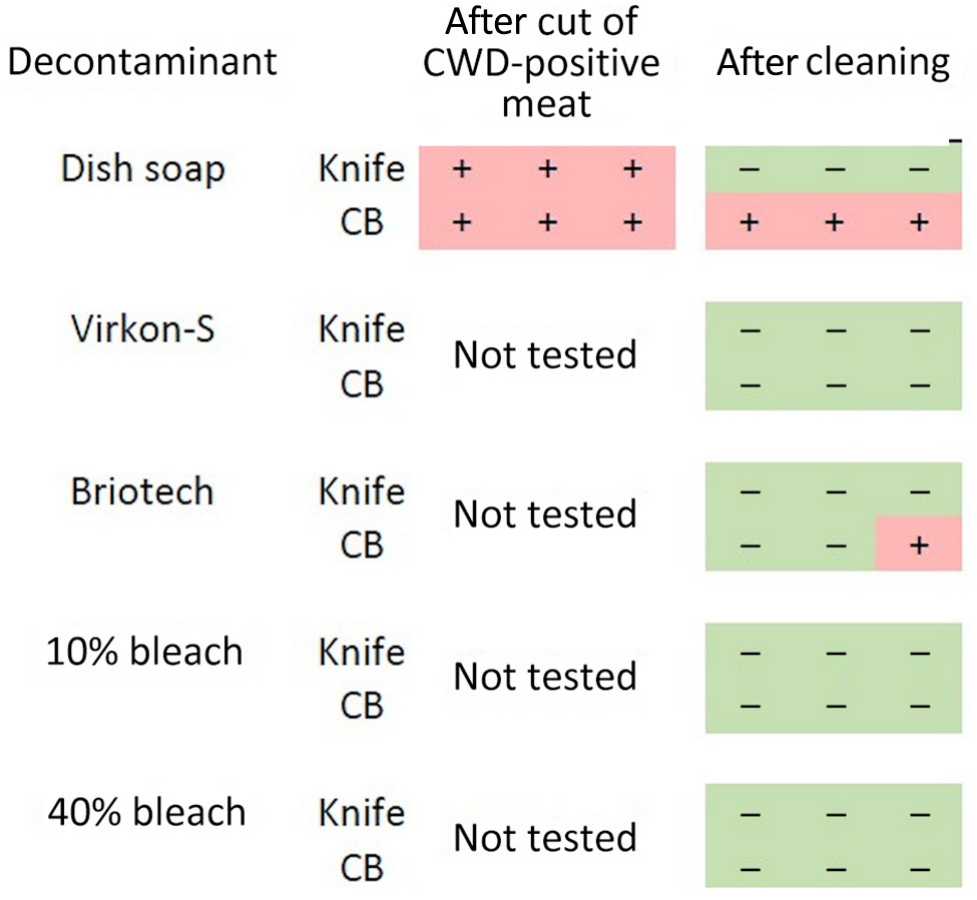
Results from knife and cutting board experiments after CWD-positive muscle cutting and after cleaning with 5 decontaminants in study of detection and decontamination of CWD prions during venison processing. Dish soap, Dawn brand (Procter & Gamble, https://dawn-dish.com); Briotech, 0.02% hypochlorous acid (https://briotechusa.shop); Virkon-S, 2% potassium peroxymonosulfate (Lanxess AG, https://lanxess.com); bleach, 10% vol/vol (7,500 ppm) and 40% vol/vol (30,000 ppm) commercial bleach solutions (7.5% sodium hypochlorite; The Clorox Company, https://www.clorox.com). CB, cutting board; CWD, chronic wasting disease.

We detected significant seeding activity on the stainless steel knife and polyethylene cutting board after cutting CWD-positive muscle (Figure 3). We also detected significant seeding activity on the cutting board after cleaning with dish soap but not on the knife or sponge after cleaning with dish soap (Figure 3; sponge data not shown).

#### Briotech, 2% Virkon-S, 10% Bleach, 40% Bleach

After cleaning the CWD-contaminated polyethylene cutting board with Briotech, we detected significant seeding activity in 1 sample (Figure 3). We detected no significant seeding activity on CWD-contaminated knives after cleaning with any of the decontaminants (Figure 3) or on CWD-contaminated cutting boards after decontamination with Virkon-S, 10% (7,500 ppm) bleach, or 40% (30,000 ppm) bleach (Figure 3).

### Meat Cross-Contamination

**Figure 4:**
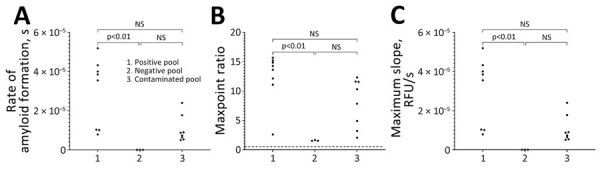
Results of real-time quaking-induced conversion in study of detection and decontamination of chronic wasting disease prions during venison processing. Results are shown for the CWD-positive muscle homogenate (positive pool), CWD-negative muscle homogenate before passing through a contaminated grinder (negative pool), and the CWD-negative muscle homogenate after passing through a contaminated meat grinder (contaminated pool). A) Rate of amyloid formation; B) maxpoint ratio (ratio of the maximum value to the initial reading) (*28*); C) maximum slope. NS, not statistically significant.

We detected an 88% significant seeding rate in the CWD-positive muscle homogenate pool (7/8 biological replicates) (Figure 4). When we passed CWD-positive muscle homogenate through a grinder, we detected significant seeding activity on the plate cutter (2/3 biological replicates, 66.7%) and screw ring thread (1/3 biological replicates, 33.3%). We detected no seeding activity on the worm spindle of the grinder. When we passed CWD-negative muscle through the grinder after the CWD-positive muscle homogenate, we found a 63% seeding activity rate (5/8 biological replicates) (Figure 4).

### Meat Grinder

**Figure 5:**
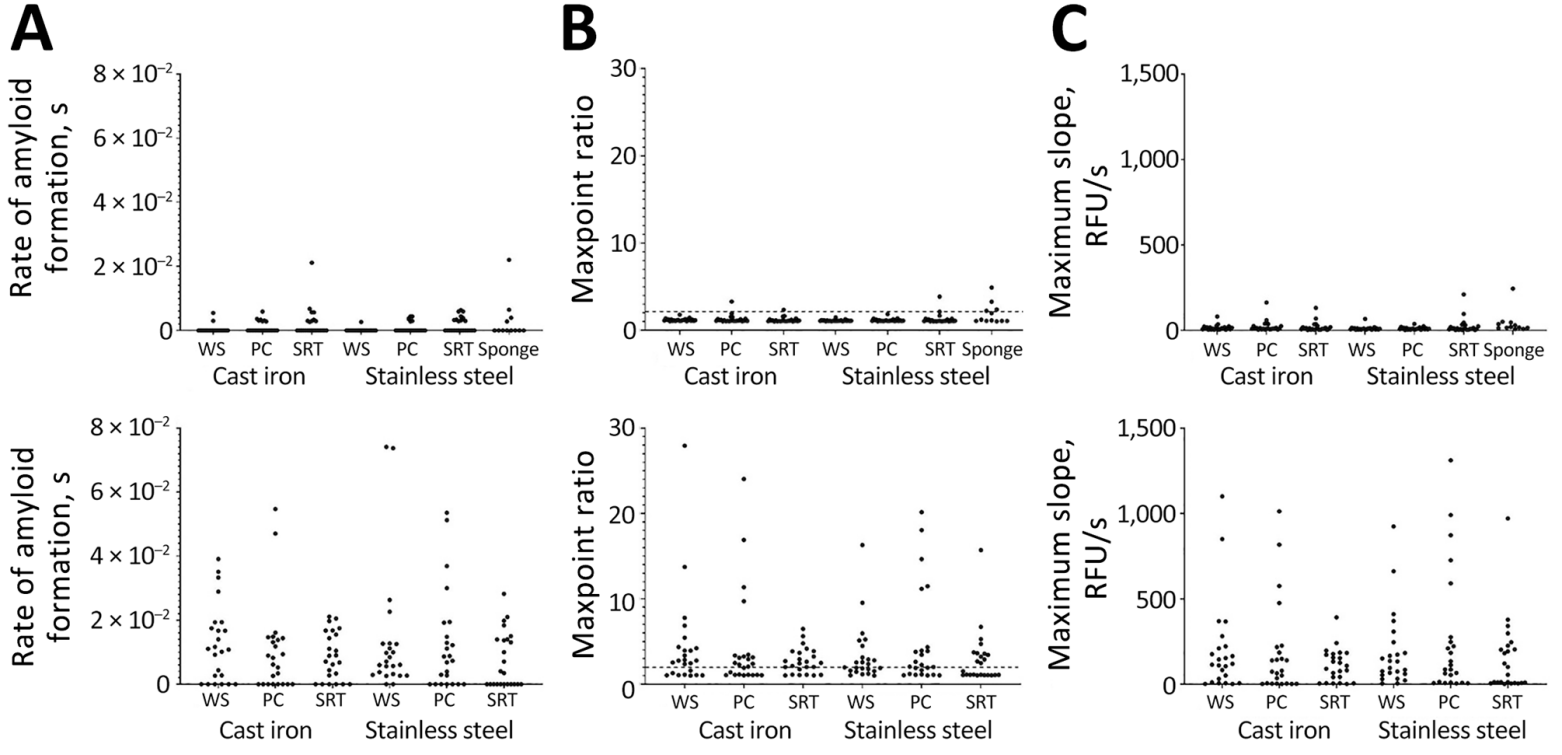
Results of real-time quaking-induced conversion conducted on meat grinder swab samples in study of detection and decontamination of chronic wasting disease prions during venison processing. A) Rate of amyloid formation; B) maxpoint ratio (ratio of the maximum value to the initial reading); C) maximum slope. Results are shown for negative controls (before meat grinder was used; top row) and after homogenate (after chronic wasting disease–positive muscle was passed through; bottom row). Each dot is an average of a single biologic replicate (consisting of 8 technical replicates). Each set of paired samples (e.g., cast iron worm spindles) resulted in a statistically significant difference post homogenate compared with the negative samples (p<0.05; data not shown). Dashed lines indicate the cutoff for significance using the maxpoint ratio (*28*). PC, plate cutter; SRT, screw ring threads; WS, worm spindle.

**Figure 6:**
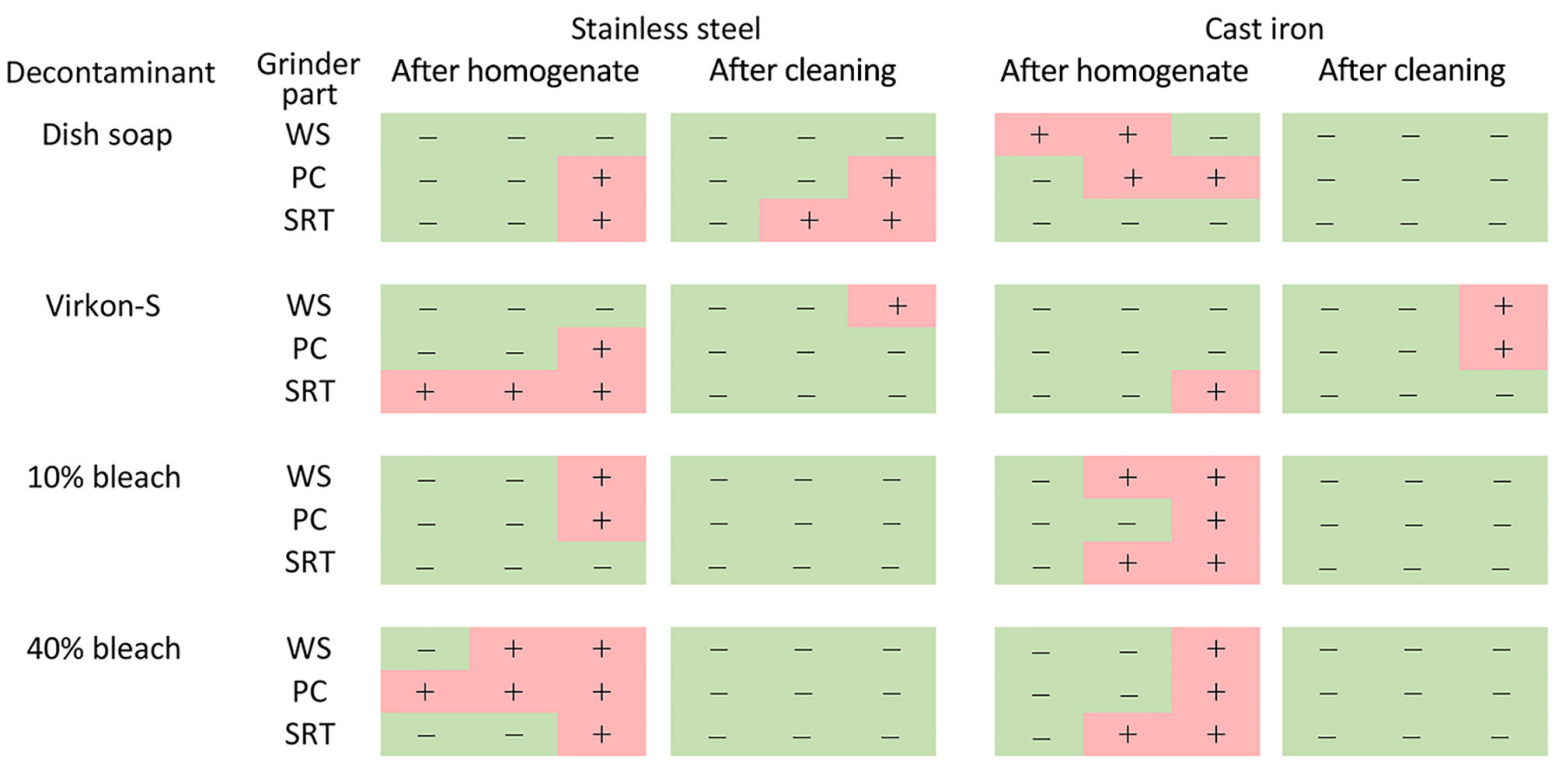
Results from stainless steel and cast iron meat grinder experiments in study of detection and decontamination of chronic wasting disease prions during venison processing. Samples were taken after chronic wasting disease–positive muscle homogenate passed through each grinder and after cleaning with each of 4 decontaminants: dish soap, Dawn brand (Procter & Gamble, https://dawn-dish.com); Virkon-S, 2% potassium peroxymonosulfate (Lanxess AG, https://lanxess.com); and bleach, 10% vol/vol (7,500 ppm) and 40% vol/vol (30,000 ppm) commercial bleach solutions (7.5% sodium hypochlorite; The Clorox Company, https://www.clorox.com). PC, plate cutter; SRT, screw ring threads; WS, worm spindle.

**Figure 7:**
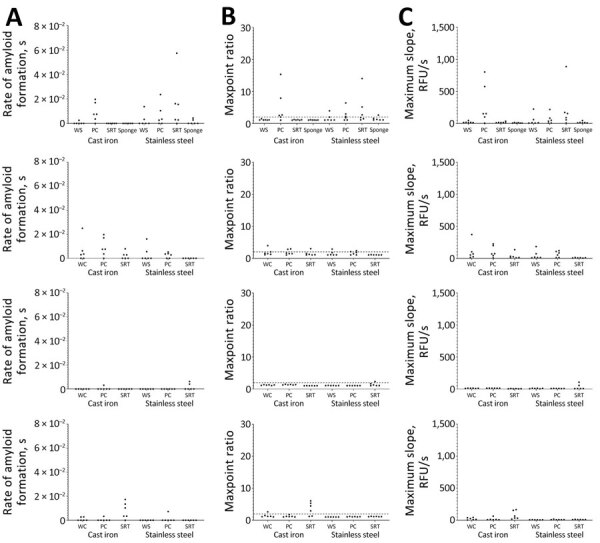
Results of real-time quaking-induced conversion assays conducted on meat grinder swab samples in study of detection and decontamination of chronic wasting disease prions during venison processing. Swab samples were taken after grinder parts were soaked in 1 of 4 decontaminants (after chronic wasting disease–positive muscle was passed through each): dish soap (top row), Dawn brand (Procter & Gamble, https://dawn-dish.com); Virkon-S (second row), 2% potassium peroxymonosulfate (Lanxess AG, https://lanxess.com); and bleach, 10% vol/vol (7,500 ppm) (third row) and 40% vol/vol (30,000 ppm) (bottom row) commercial bleach solutions (7.5% sodium hypochlorite; The Clorox Company, https://www.clorox.com). A) Rate of amyloid formation; B) maxpoint ratio (ratio of the maximum value to the initial reading); C) maximum slope. Each dot is an average of a single biological replicate (consisting of 8 technical replicates). PC, plate cutter; SRT, screw ring threads; WS, worm spindle.

All negative controls of the stainless steel and cast iron grinder parts were negative by RT-QuIC (Figure 5). Significant seeding activity was demonstrated from swabs from all stainless steel and cast iron grinder parts after passage of CWD-positive muscle homogenate (Figures 5, 6). Significant seeding activity was demonstrated from multiple swabs after decontamination of the stainless steel and cast iron grinder parts with dish soap and Virkon-S, whereas no significant seeding activity was demonstrated from swabs after decontamination with 10% (7,500 ppm) and 40% (30,000 ppm) bleach (Figures 6, 7).

## Discussion

In this study, we examined the contamination of commonly used meat-processing equipment with CWD prions from CWD-positive muscle and the efficacy of decontaminants commonly used in homes or previously shown to have variable levels of efficacy for prion decontamination (*20*–*22*). In addition, we investigated the cross-contamination of meat in CWD prion–contaminated meat-processing equipment. Using a conservative approach in determining whether samples were positive (by requiring positivity using all 3 RT-QuIC metrics), we found that CWD prion seeding activity can be detected on common meat-processing surfaces after coming into contact with CWD-positive white-tailed deer muscle; CWD prion seeding activity can be transferred to CWD-negative meat after passing through a contaminated meat grinder; Virkon S, 10% bleach, and 40% bleach were effective for CWD prion decontamination on surfaces, as shown by removal of seeding activity; and surface composition might play a role in CWD prion detection and decontamination on meat-processing equipment.

On the basis of our results, CWD prion seeding activity is clearly detectable on common meat-processing equipment, such as knives, polyethylene cutting boards, and multiple parts of meat grinders. We would note that the presence of seeding activity does not directly imply infectivity, and the relationship between seeding activity and infectivity can be complicated. However, given that seeding activity is correlated with infectivity (*29*), and we are using materials that have verification of PrP^Sc^ presence by other means, the presence or absence of seeding activity in our study can be reasonably be concluded to correlate with the presence or absence of infectivity. Further investigation is warranted into the contamination regimes and decontaminants described using animal bioassays.

Of note, seeding activity was demonstrated not only in tissues containing high levels of prions (i.e., brain from a CWD-positive animal) but also in muscles with progressively lower levels of prions from a clinical deer and 2 hunter-harvested deer. We also detected seeding activity in the CWD-negative muscle homogenate after it passed through the contaminated meat grinder. Those findings exemplify real-life scenarios with implications for the food supply in which surfaces and tools are reused between deer and meat is mixed from multiple deer, potentially with nonclinical, untested deer.

Except for dish soap, all of the decontamination agents used in this study have been shown to inactivate prions (*20*–*22*); the active ingredient in Briotech is hypochlorous acid (*22*) and the active ingredient in Virkon-S is peroxymonosulfate (*20*). We found that 10% bleach and 40% bleach were highly effective at decontaminating the meat-processing surfaces tested in this study; Virkon-S was slightly less effective. Briotech was less effective and inconsistent in decontamination. Although the overall findings are promising, further investigation is warranted, especially given the lower relative prion load in our CWD-positive muscle pool used in the meat grinder studies. Decontamination efficacy on grinder parts could be further investigated by using tissues with prion loads higher than that typical in muscle. Reduced concentrations of bleach and contact times could still result in effective decontamination, but we did not test the lowest effective duration or concentration of decontaminants, nor did we test how repeated disinfection with the solutions affects surface integrity and ongoing disinfection efficacy. This factor is noteworthy because the chemicals can compromise the integrity of surfaces such as stainless steel, leading to unknown consequences of prion adsorption and decontamination. In addition, although we observed a few negative control swabs of grinder parts that were positive on initial test but negative upon retest, we hypothesize that those interferences arose from grease or metallic debris from the machining of parts. From a cleaning protocol perspective, the scrubbing of tools, parts, and surfaces is a key step, because tissue debris tends to remain on some surfaces after a decontamination soak. As demonstrated by others (*21*), decontaminants are ineffective at penetrating tissue, so removal of the tissue remnants will lead to more effective decontamination and reduce cross-contamination. This factor is one plausible explanation for the reduced efficacy (i.e., retained seeding activity) of Virkon-S in one of the treatments. Containing and disposing of the predecontamination cleaning wash is also a consideration for environmental prion contamination.

Of note, we saw differences in the detection of prion seeding activity between the knife and cutting board; after decontamination with dish soap, we were able to detect prion seeding on the surface of the cutting board but not on the surface of the knife. Curiously, we did not detect prion seeding activity in any of the sponge samples. We cannot discount the possibility that prions were removed from the knife surface by dish soap and simply remained in the decontaminant solution, speaking back to our point regarding containment and disposal of the cleaning solution itself. The principal components of dish soap are surfactants (*30*), which can remove adsorbed prions from surfaces, resulting in a lack of detection on the steel surface while also diluting prions to the point that they are undetectable by RT-QuIC. Those factors must all be considered when planning and implementing cleaning and decontamination of environmental prions.

In conclusion, our results show that processing of CWD-positive cervids for consumption has the potential to contaminate meat-processing equipment and cross-contaminate downstream meat products. Further, our data and previous studies (*20*,*21*) indicate that Virkon-S and bleach solutions with appropriate contact times (as little as 5 minutes) can effectively decontaminate nonporous surfaces of CWD prions. After the bovine spongiform encephalopathy outbreak in Europe in the early 1990s, processing practices became mandatory to remove and avoid specified risk materials (e.g., spinal cord, brain) and incorporate downstream molecular testing procedures to identify contamination in meat products (*31*). Our findings indicate similar practices may be necessary to reduce CWD exposure in humans through meat processing. However, given the unique features of CWD prions, contamination of equipment and surfaces is more challenging to control. This concern further highlights the importance of testing cervids before processing, in addition to the need for compliance with effective meat processing, environmental screening, waste management, and cleaning and decontamination protocols.

This article was published as a preprint at https://www.biorxiv.org/content/10.1101/2024.07.23.604851v1.

Appendix Additional information about detection and decontamination of chronic wasting disease prions during venison processing.
